# A survey of microRNA single nucleotide polymorphisms identifies novel breast cancer susceptibility loci in a case-control, population-based study of African-American women

**DOI:** 10.1186/s13058-018-0964-4

**Published:** 2018-06-05

**Authors:** Jeannette T. Bensen, Mariaelisa Graff, Kristin L. Young, Praveen Sethupathy, Joel Parker, Chad V. Pecot, Kevin Currin, Stephen A. Haddad, Edward A. Ruiz-Narváez, Christopher A. Haiman, Chi-Chen Hong, Lara E. Sucheston-Campbell, Qianqian Zhu, Song Liu, Song Yao, Elisa V. Bandera, Lynn Rosenberg, Kathryn L. Lunetta, Christine B. Ambrosone, Julie R. Palmer, Melissa A. Troester, Andrew F. Olshan

**Affiliations:** 1 Department of Epidemiology, Gillings School of Global Public Health, University of North Carolina at Chapel Hill, Chapel Hill, NC 27599 USA; 2 Department of Biomedical Sciences, College of Veterinary Medicine, Cornell University, Ithaca, NY 14853 USA; 3 Department of Genetics, University of North Carolina at Chapel Hill, Chapel Hill, NC 27599 USA; 4 Department of Medicine, Division of Oncology, School of Medicine, University of North Carolina at Chapel Hill, Chapel Hill, NC 27599 USA; 5 Biological and Biomedical Sciences Program, University of North Carolina at Chapel Hill, Chapel Hill, NC 27599 USA; 6 Slone Epidemiology Center at Boston University, Boston, MA 02215 USA; 7 Department of Nutritional Sciences, University of Michigan School of Public Health, Ann Arbor, MI 48109 USA; 8 Department of Preventive Medicine, Keck School of Medicine, University of Southern California/Norris Comprehensive Cancer Center, Los Angeles, CA 90033 USA; 9 Department of Cancer Prevention and Control, Roswell Park Cancer Institute, Buffalo, NY 14263 USA; 10 Department of Biostatistics and Bioinformatics, Roswell Park Cancer Institute, Buffalo, NY 14263 USA; 11 Cancer Prevention and Control, Rutgers Cancer Institute of New Jersey, New Brunswick, NJ 08903 USA; 12 Department of Biostatistics, Boston University School of Public Health, Boston, MA 02118 USA

**Keywords:** microRNA, miRNA, SNP, Breast cancer, African American, Case-control

## Abstract

**Background:**

MicroRNAs (miRNAs) regulate gene expression and influence cancer. Primary transcripts of miRNAs (pri-miRNAs) are poorly annotated and little is known about the role of germline variation in miRNA genes and breast cancer (BC). We sought to identify germline miRNA variants associated with BC risk and tumor subtype among African-American (AA) women.

**Methods:**

Under the African American Breast Cancer Epidemiology and Risk (AMBER) Consortium, genotyping and imputed data from four studies on BC in AA women were combined into a final dataset containing 224,188 miRNA gene single nucleotide polymorphisms (SNPs) for 8350 women: 3663 cases and 4687 controls. The primary miRNA sequence was identified for 566 miRNA genes expressed in Encyclopedia of DNA Elements (ENCODE) Tier 1 cell types and human pancreatic islets. Association analysis was conducted using logistic regression for BC status overall and by tumor subtype.

**Results:**

A novel BC signal was localized to an 8.6-kb region of 17q25.3 by four SNPs (rs9913477, rs1428882938, rs28585511, and rs7502931) and remained statistically significant after multiple test correction (odds ratio (OR) = 1.44, 95% confidence interval (CI) = 1.26–1.65; *p* = 3.15 × 10^−7^; false discovery rate (FDR) = 0.03). These SNPs reside in a genomic location that includes both the predicted primary transcript of the noncoding miRNA gene *MIR3065* and the first intron of the gene for brain-specific angiogenesis inhibitor 1-associated protein 2 (*BAIAP2*)*.* Furthermore, miRNA-associated SNPs on chromosomes 1p32.3, 5q32, and 3p25.1 were the strongest signals for hormone receptor, luminal versus basal-like, and HER2 enrichment status, respectively. A second phase of genotyping (1397 BC cases, 2418 controls) that included two SNPs in the 8.6-kb region was used for validation and meta-analysis. While neither rs4969239 nor rs9913477 was validated, when meta-analyzed with the original dataset their association with BC remained directionally consistent (OR = 1.29, 95% CI = 1.16–1.44 (*p* = 4.18 × 10^–6^) and OR = 1.33, 95% CI = 1.17–1.51 (*p* = 1.6 × 10^–5^), respectively).

**Conclusion:**

Germline genetic variation indicates that *MIR3065* may play an important role in BC development and heterogeneity among AA women. Further investigation to determine the potential functional effects of these SNPs is warranted. This study contributes to our understanding of BC risk in AA women and highlights the complexity in evaluating variation in gene-dense regions of the human genome.

**Electronic supplementary material:**

The online version of this article (10.1186/s13058-018-0964-4) contains supplementary material, which is available to authorized users.

## Background

MicroRNAs (miRNAs) are small noncoding RNAs that were formally recognized in 2001 [1] as one of the largest classes of gene regulators in eukaryotes [2]. miRNAs undergo a complex, multistep process of biogenesis summarized by Lin and Gregory in 2015 [3]. Briefly, within the nucleus, a primary miRNA transcript (pri-miRNA)—usually several hundred nucleotides (nt) to greater than 1 megabase (Mb) in length—is cleaved to create a precursor miRNA (pre-miRNA) approximately 70 nt in length which folds to form a stem-loop intermediate. This intermediate is exported from the nucleus and further processed to a miRNA duplex, approximately 22 nt in length. One strand of the miRNA duplex is loaded onto the RNA-induced silencing complex (RISC) to form a functional mature miRNA. Cleavage and processing of the pri- and pre-miRNA require sequence and secondary structure recognition by several RNA-binding proteins and their partners. Approximately 30% of mature miRNAs are processed from introns or exons of coding genes, while the remaining miRNAs are intergenic and expressed from independent transcription units. Mature miRNAs bind to the 3’ untranslated region (UTR) of target genes to silence them by either translational repression or messenger RNA (mRNA) degradation [4]. There are over 2500 identified human miRNAs [5] and each may bind to hundreds or even thousands of different target genes, coordinating expression of a large number of mRNAs; this makes them key players in gene regulatory networks [3].

miRNAs have been shown to influence numerous molecular pathways and pathological conditions, including cancer [3, 6–10], and can function as both oncogenes and tumor suppressors depending on the context. Furthermore, oncoproteins such as MYC bind to the promoters of key miRNAs, activating oncogenic miRNAs (oncomiRs) and downregulating tumor suppressor miRNAs [11–13]. In breast cancer (OMIM #114480), miRNAs have been implicated in the regulation of genes involved in pathways critically relevant to disease etiology and severity including apoptosis, cell cycle checkpoints, cell migration, invasion, and metastasis [14–17]. To a large extent, the miRNA repertoire that is present in normal and paired tumor tissue from the same organ is quite similar; however, specific miRNAs are often aberrantly elevated or suppressed in the tumor [18]. In 2011, Persson et al. performed one of the first comprehensive characterizations via next-generation sequencing (NGS) of miRNAs in paired normal and tumor breast tissue and identified 361 new miRNAs [18]. While the functionality of some of the miRNAs identified by deep sequencing remains unknown, about two-thirds of these newly identified miRNAs were expressed in other tissues, and nearly half were associated with components of the RISC and were found in estrogen receptor-positive, invasive breast ductal carcinoma cells. Germline single nucleotide polymorphisms (SNPs) in critical regions of miRNA genes including the promoter and primary transcripts may contribute to the dysregulation in miRNA biogenesis and expression differences common in breast cancer.

Over the last decade there has been tremendous progress made in the field of miRNAs and cancer, particularly centered on miRNA expression patterns that are emerging as promising diagnostic tools and predictive markers because of their correlation with cancer progression and patient survival [3]. However, little is known about the role of germline variation in miRNAs and susceptibility to cancer.

Currently, known germline genetic variation primarily from studies of European women explains only 50% of the familial aggregation of breast cancer (BC), suggesting that numerous other susceptibility gene variants have yet to be uncovered [19]. Several molecular epidemiologic studies have assessed the association of common germline miRNA gene variation in mature and precursor miRNA sequences with disease risk, including BC [20–29]. Few epidemiologic studies have evaluated the association between a large number of germline genetic variants in the promoter and primary sequences of miRNAs and BC risk, particularly among African-American (AA) women. We sought to identify large numbers of germline miRNA gene variants associated with BC risk and subtype among women participating in a large AA BC consortium.

## Methods

### Study population

This research was conducted using data from the African American Breast Cancer Epidemiology and Risk (AMBER) Consortium, a collaboration of two case-control studies of BC in AA women (the Carolina Breast Cancer Study (CBCS) [30] and the Women’s Circle of Health Study (WCHS) [31, 32]) and two cohort studies (the Black Women’s Health Study (BWHS) [33] and the Multiethnic Cohort (MEC) [34]). AMBER has been described previously [35]. All study participants provided written informed consent and all studies obtained Institutional Review Board approval.

This analysis utilizes data from 3663 cases and 4687 controls in AMBER who provided either blood or saliva for DNA analysis. For the case-control studies, controls were identified either through Division of Motor Vehicles lists (age < 65 years) and Health Care Financing Administration lists (age ≥ 65) (CBCS), or random digit dialing and community controls (WCHS). For BWHS and MEC, controls were chosen from among women without BC, and were frequency matched to cases on geographical region, sex, race, and 5-year age group. Eligible cases were AA women with incident invasive BC or ductal carcinoma in situ (DCIS). Estrogen receptor (ER), progesterone receptor (PR), epidermal growth factor receptor 2 (HER2) receptor, and invasive status for cases was determined using pathology data from hospital or cancer registry records.

### Genotyping and quality control (QC)

Genotyping of DNA from participants in the BWHS, CBCS, and WCHS was performed by the Center for Inherited Disease Research (CIDR) using the Illumina Human Exome BeadChip v1.1. This array includes > 200,000 coding variants, as well as tag SNPs for genome-wide association study (GWAS) hits, a grid of common variants, and ancestry informative markers (AIMs). A description of the exome chip design is available from http://genome.sph.umich.edu/wiki/Exome_Chip_Design. In addition to the standard BeadChip, the chip included approximately 159,000 SNPs of custom content focused on BC pathways (e.g., steroid hormone metabolism, insulin and insulin-like growth factors, inflammatory and immune factors, and vitamin D).

A total of 405,555 SNPs were genotyped, and 300,008 SNPs remained after excluding variants that failed technical filters imposed by CIDR, or QC filters recommended by the University of Washington. Briefly, genotypes with a GenCall score < 0.15 were classified as missing, and SNPs were removed if they were monomorphic, had poor cluster properties (ex. cluster separation < 0.2 or < 0.3 depending on allele frequency), call rates < 0.98, Hardy-Weinberg Equilibrium *p* < 1 × 10^−4^, > 1 Mendelian error in trios from HapMap, or > 2 discordant calls in duplicate samples. Mitochondrial and Y chromosome SNPs were also removed. Genotypes were attempted for 6936 participants from the BWHS, CBCS, and WCHS, and were completed with a call rate > 98% for 6828 participants, which included 3130 cases (963 ER negative, 1674 ER positive, 493 ER unknown) and 3698 controls. Imputation was performed by the University of Washington using the IMPUTE2 software [36] and the 1000 Genomes Phase I reference panel (5/21/2011 1000 Genomes data, December 2013 haplotype release).

Genetic data from 533 cases (135 ER negative, 309 ER positive, and 89 ER unknown) and 989 controls in the MEC were available from a previous GWAS on the Illumina Human 1 M-Duochip [37]. SNPs from MEC were imputed to the same release of 1000 Genomes and combined with the genotype data from the Illumina Human Exome BeadChip v1.1. Additional exclusion criteria applied to the four-study merged dataset were: variants with mismatching alleles or allele frequencies that were different by more than 0.15 in MEC when compared with the other three studies; variants with allele frequencies < 0.5%; and variants with imputation score INFO < 0.5 in either MEC or any of the other three studies. The final merged dataset included genotypes from 8350 women, 3663 cases (1983 ER positive, 1098 ER negative, 582 unknown), and 4687 controls.

### miRNA annotation, SNP selection and QC

Among the genotyped and imputed SNPs, miRNA variants were defined as those within promoter, pri-miRNA, pre-miRNA, mature, or downstream regions of a known human miRNA. Mature and pre-miRNA sequence locations were identified from the miRNA database, miRBase release 21 [5, 38] . Pri-miRNAs were identified by integrative analysis of chromatin immunoprecipitation and massively parallel DNA sequencing (ChIP-seq) data from the Encyclopedia of DNA Elements (ENCODE) project using an algorithm described previously [39]. Five hundred and sixty six miRNA genes with pri-miRNA sequence expressed in six cell lines and tissue types (all ENCODE Tier 1 cell types plus human pancreatic islets) were the focus of this analysis. We extended the pri-miRNA 5 kilobases (kb) upstream of the 5′-end (putative promoter) and 1 kb downstream of the 3′-end (additional putative regulatory region). Variants that could be defined as having multiple miRNA locations were defined by their most unique location with the following priority: mature > precursor > primary > promoter > downstream. For example, a variant in the mature miRNA sequence is also by default in the pri-miRNA; however, according to our prioritization it would be defined as a mature miRNA sequence variant. SNPs were restricted to those variants with minor allele frequencies (MAF) ≥ 1%. Annotation defined a total of 224,188 miRNA gene SNPs, with MAF ≥ 1%, from the following miRNA gene regions: 10,435 promoter, 182,593 primary, 272 precursor, 158 mature, and 2150 downstream variants. The impact of genotype platform was evaluated by quantile-quantile plots both with and without MEC genotypes, both yielding a lambda = 0.991.

### Association analysis

Single variant analyses were conducted using logistic regression as implemented in PLINK version 1.07. Models were adjusted for age group (by ~ 10-year intervals), study site, geographic group of residence, DNA source, and ancestry by including principal components 5, 6, and 8 in the model given their association with BC at *p* < 0.1 [40]. Models were run for all cases versus all controls and for all hormone receptor subtyped (ER, PR, and HER2) cases versus controls, respectively. Additional models were run for case-only subtype analyses (*n* = 3663, eligible cases with biomarker and covariate information) using ER, PR, and HER2 receptor marker status. Specifically, the following three case-only subtype analyses were performed: 1) hormone receptor positive (ER positive or PR positive, *n* = 2081) versus hormone receptor negative (ER negative and PR negative, *n* = 997); 2) luminal (ER positive or PR positive, *n* = 1613) versus basal-like (ER negative, PR negative, and HER2 negative, *n* = 405) [41]; and 3) HER2 enriched (*n* = 1356) versus HER2 negative (*n* = 344). *P* values were corrected within subtype analyses for multiple comparisons using the false discovery rate (FDR) at 5% [42]. In all analyses, both invasive and in situ cases were combined.

### Validation and meta-analysis

A second phase of genotyping (1397 BC cases, 2418 controls) conducted in three of the four studies within AMBER (CBCS, WCHS, and BWHS) on the Illumina’s Infinium Multi-Ethnic Genotyping Array (MEGA) Chip that included study-specific content and SNPs rs4969239 and rs9913477 was used for validation and meta-analysis. Similar to the association analysis, logistic regression implemented in PLINK version 1.07 was used and models were adjusted for age group (by ~ 10-year intervals), study site, DNA source, and ancestry by including principal component 1 in the model given its association with BC at *p* < 0.1. Validation for each variant was evaluated for directional consistency and tested at the *p* < 0.05 level. In the meta-analysis, both the original and the second phase of genotyping were combined and the *p* value corrected for multiple comparisons using an FDR at 5% [42].

Power was calculated for detecting an odds ratio (OR) of 1.44 and an OR of 1.30 (a 10% reduction in effect estimate assuming the original OR is an overestimate of the actual effect) using a two-sided, *p* = 0.05 significance, log-additive mode of inheritance, allele frequency of 0.06 (the same as rs9913477 MAF in the study population), control to case ratio of 1.7 with 1397 cases, and prevalence of disease of 10%.

## Results

Table 1 provides a distribution of key characteristics of the study population by case or control status and includes age at diagnosis, study site, DNA source, as well as clinical parameters (tumor stage and receptor status). The study population originates from a broad geographical region of the United States with most cases from the Northeast and South. Overall, the vast majority of the cases have known ER or PR receptor status; however, over half do not have known HER2 receptor status. Among cases with known receptor status for all three markers, approximately 20% are triple negative.

**Table 1:** Characteristics of the study population

	Controls (*n* = 4687)		Cases (*n* = 3663)	
	Frequency	Mean (SD) or %	Frequency	Mean (SD) or %
Age at enrollment (years)	4687	55.62 (12.01)	3663	54.94 (11.74)
Age at enrollment (years)				
18–29	24	0.51	30	0.82
30–39	396	8.45	306	8.35
40–49	1107	23.62	945	25.8
50–59	1461	31.17	1087	29.68
60–69	986	21.04	819	22.36
70–79	609	12.99	433	11.82
80+	104	2.22	43	1.17
DNA source				
Blood	1817	38.77	1961	53.54
Mouthwash	2243	47.86	853	23.29
Saliva	627	13.38	849	23.18
Study				
BWHS	2249	48.98	901	24.6
WCHS	834	17.79	821	22.41
CBCS	615	13.12	1408	38.44
MEC	989	21.1	533	14.55
Location				
New Jersey (NJ)	573	12.23	613	16.73
Northeast (except NJ)	1245	26.56	441	12.04
South	1476	31.49	1720	46.96
Midwest	238	5.08	200	5.46
West	1155	24.64	689	18.81
Stage				
In situ	NA		376	10.26
Invasive	NA		2528	69.01
Unknown	NA		759	20.72
Tumor receptor status				
ER				
Positive	NA		1983	54.14
Negative	NA		1098	29.98
Unknown	NA		582	15.89
PR				
Positive	NA		1580	43.13
Negative	NA		1343	36.66
Unknown	NA		740	20.2
HER2				
Positive	NA		344	9.39
Negative	NA		1356	37.02
Unknown	NA		1963	53.59
Triple negative				
Yes	NA		405	11.06
No	NA		1613	44.03
Unknown	NA		1645	44.91

BWHS, Black Women’s Health Study; CBCS, Carolina Breast Cancer Study; ER, estrogen receptor; HER2, human epidermal growth factor receptor 2; MEC, Multiethnic Cohort; NA, not applicable; PR, progesterone receptor; SD, standard deviation; WCHS, Women’s Circle of Health Study

### Genomic location of novel miRNA SNPs associated with BC in African-American women: case-control analysis

**Fig. 1:**
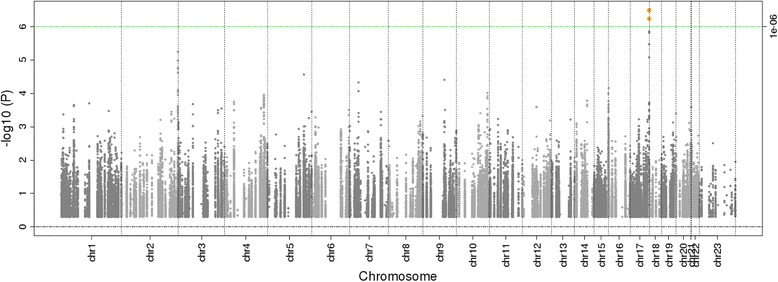
Manhattan plot of miRNA SNP and breast cancer risk in the four-site AMBER Consortium (*n* = 8350, with 3663 cases and 4687 controls). The green line represents a significant *p* value threshold of 1 × 10^−6^ at a false discovery rate (FDR) of 5%

The main case-control association analysis identified seven SNPs (five imputed and two genotyped) in a 16.5-kb region on chromosome 17q25.3 (Fig. 1 and Table 2), with imputed rs9913477 (INFO *r*^2^ = 0.99; MAF = 0.06; OR = 1.44, 95% confidence interval (CI) = 1.26–1.65; *p* = 3.15 × 10^−7^; FDR = 0.03) emerging as the top hit. Following FDR correction, four of the seven remained significantly associated with BC risk, spanning an 8.6-kb region (Table 2). All four SNPs reside in a genomic region that includes the first intron of the brain-specific angiogenesis inhibitor 1-associated protein 2 (*BAIAP2*), as well as the predicted primary transcript for *MIR3065*. Linkage disequilibrium (LD) between the top hit (rs9913477) and the other three statistically significant SNPs was high (*r*^2^ = 0.94) for two (rs1428882938 and rs28585511) and perfect (*r*^2^ = 1.0) for the third (rs7502931), suggesting they are all tagging the same signal in this population. Subsequently, ER/PR subtype analysis was conducted for all seven SNPs with *p* < 5 × 10^–6^ in the full analysis (Additional file 1: Table S1) and identified that the signal and pattern of association in this region was statistically significant based on FDR in ER^+^ versus controls, most likely because it had the largest sample size. While the other subtype analyses versus controls were not significant, the magnitude of the odds ratio was similar to that observed in ER^+^ versus controls. However, when we look at the case-only subtype analyses (e.g., ER^+^ versus ER^–^, PR^+^ versus PR^–^) we see a reduction in the magnitude of the odds ratio suggesting that this region is more likely to be associated generally with the development of breast cancer rather than a particular subtype. Additionally, in a subanalysis of ER positive cases (*n* = 1983) versus controls (*n* = 4687) and PR positive cases (*n* = 1580) versus controls (*n* = 4687) the same 17q25.3 locus top hit (rs9913477) emerged, but was statistically significantly associated with BC after FDR correction only for the largest subgroup of ER positive cases (INFO *r*^2^ = 0.99, MAF = 0.58; OR = 1.53, 95% CI = 1.30–1.81; *p* = 4.29 × 10^–7^; FDR = 0.027). The variant rs9913477 was also the second most significant SNP in the ER positive plus PR-positive case group versus control analysis but did not reach statistical significance after FDR correction (data not shown). In the ER negative, PR-negative, and ER negative plus PR-negative cases versus control analysis, rs80339298 located in the primary sequence of *MIR761* on chromosome 1 emerged as the top SNP but did not reach statistical significance after FDR correction (data not shown).

**Table 2:** Association of the top seven miRNA SNPs with *p* < 5 × 10^−6^ and breast cancer risk

SNP	Chromosome:position^a^	Effect/other allele	EAF	OR (95% CI)	*p* ^b^	FDR *p*^c^	INFO (*r*^2^)
rs142882938	17:79010031	C/CT	0.06	1.45 (1.24–1.70)	5.9 × 10^–7^	**0.03**	0.97
rs4969239^d^	17:79010544	G/A	0.08	1.35 (1.20–1.52)	1.4 × 10^–6^	0.06	–
rs28585511	17:79010609	T/A	0.06	1.45 (1.26–1.66)	5.8 × 10^–7^	**0.03**	0.98
rs4969351	17:79011141	A/G	0.08	1.35 (1.20–1.52)	3.4 × 10^–6^	0.11	0.99
rs9913477	17:79015698	G/A	0.06	1.44 (1.26–1.65)	3.2 × 10^–7^	**0.03**	0.99
rs7502931	17:79018677	G/A	0.06	1.44 (1.26–1.65)	3.4 × 10^–7^	**0.03**	0.99
rs4969366^d^	17:79026572	G/A	0.05	1.45 (1.24–1.70)	1.5 × 10^–6^	0.06	–

The seven single nucleotide polymorphisms (SNPs) are intronic to BAIAP2 and located in the primary transcript of miR-3065

Significant FDR results are shown in bold

CI, confidence interval; EAF, effect allele frequency; FDR, false discovery rate; INFO, imputation quality score; OR, odds ratio

^a^Human Genome GRCh37/hg19 assembly, NT_010783.15

^b^Additive genetic models were adjusted for age group (by ~ 10-year intervals), study site, geographic region of residence, DNA source, and ancestry (PCs 5, 6, and 8 associated with cancer trait, *p* < 0.1). Sample size: 3663 cases and 4687 controls

^c^Adjustment for multiple comparisons using the FDR

^d^Genotyped SNPs, with the other SNPs having been imputed to 1000 Genome Project data

### Case-only subtype analysis

Top SNPs identified in each of the three subtype analyses are provided in Table 3. These top SNPs were located on chromosomes 1p32.3 (rs80339298, *OSBPL9* intron 11, NT_032977.10), 5q32 (rs147821319, *PPARGC1B* intron 7, NM_001172698), and 3p25.1 (rs116367195, intergenic between *BTD* and *ANKRD2,* NM_001195099) from the GRCh38.p2 assembly for hormone receptor, luminal versus basal-like, and HER2 enrichment status, respectively. All three SNPs were low frequency (MAF < 5%) and none were statistically significant after FDR correction.

**Table 3:** Top SNP hits for breast cancer subtype analyses

Breast Cancer Subtype	Hormone Receptor +/−	Luminal / Basal-like	HER2 +/−
Sample size	2081/997	1613/405	1356/344
SNP ID	rs80339298	rs147821319	rs116367195
Chromosome:Position*	1:52244019	5:149217038	3:15693446
Effect/Other	A/G	A/G	G/A
Reference Sequence	NT_032977.10	NM_001172698	NM_001195099
OR (95%CI)	2.11 (1.54, 2.89)	2.20 (1.52, 3.19)	2.70 (1.72, 4.24)
EAF	0.02	0.04	0.97
*p*-value**	2.90 × 10^−6^	2.34 × 10^−5^	1.59 × 10^−5^
FDR *p*-value***	0.16	0.37	0.84

Abbreviations: OR: odds ratio; 95% CI of the OR; EAF: effect allele frequency; FDR: false discovery rate

* Chromosome: position from GRCh37/hg19 Assembly

** Additive genetic model was adjusted for age group (by ~ 10 year intervals), study site, geographic region of residence, DNA source, and ancestry (PCs 5, 6 and 8 - associated with cancer trait, *p*-value< 0.1)

***Adjustment for multiple comparisons using the False Discovery Rate (FDR) within each subtype analysis

### Validation and meta-analysis

A stage 2 analysis of rs4969239 (OR = 1.07, 95% CI = 0.83–1.39; p = 5.78 × 10^–1^ and rs9913477 (OR = 0.86, 95% CI = 0.62–1.18; *p* = 3.56 × 10^–1^) failed to validate their association with BC at a nominal *p* value. However, when meta-analyzed with the original dataset, the association of rs4969239 (OR = 1.29, 95% CI = 1.16–1.44); *p* = 4.18 × 10^–6^) and rs9913477 (OR = 1.33, 95% CI = 1.17–1.51; *p* = 1.60 × 10^–5^) with BC remained directionally consistent (Table 4).

**Table 4:** Stages 1 and 2 and meta-analysis of rs9913477 and rs4969239 located in the primary transcript of miR-3065

SNP	Chromosome:Position^a^	Effect/other allele	Stage^b^	Sample size	EAF	OR (95% CI)	*p*
rs4969239	17:79010544	G/A	Stage 1	8350	0.08	1.35 (1.2–1.52)	1.40 × 10^–6^
			Stage 2	3814	0.08	1.07 (0.83–1.39)	5.78 × 10^–1^
			Meta-analysis	12,164	0.08	1.29 (1.16–1.44)	4.18 × 10^–6^
rs9913477	17:79015698	G/A	Stage 1	8350	0.06	1.44 (1.30–1.58)	3.15 × 10^–7^
			Stage 2	3815	0.06	0.86 (0.62–1.18)	3.56 × 10^–1^
			Meta-analysis	12,165	0.06	1.33 (1.17–1.51)	1.60 × 10^–5^

CI, confidence interval; EAF, effect allele frequency; OR, odds ratio; SNP, single nucleotide polymorphism

^a^Human Genome GRCh37/hg19 assembly, NT_010783.15

^b^Stage 1 model: Additive genetic models were adjusted for age group (by ~ 10-year intervals), WCHS study site, geographic region of residence, DNA source, and ancestry (PCs 5, 6 and 8 associated with cancer trait, *p* < 0.1); Stage 2 model: Additive genetic models were adjusted for age group (by ~ 10-year intervals), DNA source, and ancestry (PCs 1); Meta-analysis was performed in METAL [67]; heterogeneity *I*^2^ = 88.1 for rs9913477 and 58.1 for rs4969239

Power calculations for the detection of a SNP associated with BC at ORs of 1.44 and 1.30 at a significance of *p* ≤ 0.05 indicated that validation among the study set undergoing the second phase of genotyping was 97% and 77%, respectively.

## Discussion

In a combined analysis of four large studies of BC in AA women, we identified and annotated a novel genomic region on chromosome 17q25.3 significantly associated with BC and extended its functional interpretation with a comprehensive evaluation of miRNA gene sequence. Specifically, we have localized the BC association signal to an 8.6-kb region on chromosome 17 marked by four tightly linked, significantly associated SNPs, with rs9913477 demonstrating the strongest association. Using a second phase of genotyping we were unable to validate the association of either rs4969239 or rs9913477 with BC; however, in a meta-analysis these SNPs remained directionally consistent (OR = 1.29 and 1.33, respectively). Power calculations indicate that the validation analysis was well powered (97%) at an OR of 1.44 (our original finding and likely an overestimate of effect size) and slightly underpowered (77%) at an OR of 1.3, which represents an effect estimate 10% less that the original OR. No statistically significant miRNA SNP associations were identified in the subanalyses of hormone receptor-negative tumors. Additionally, a case-only analysis that encompassed comparisons of hormone receptor status, luminal versus basal-like subtypes, and HER2 enrichment status did not identify any statistically significant associations with BC after FDR correction. The most strongly associated miRNA-associated SNPs for each subtype analysis identified regions on chromosomes 1p32.3, 5q32, and 3p25.1, respectively. None of these regions or SNPs have been previously implicated in BC GWAS according to the GWAS Catalog (release dated 12 June 2016) [43].

**Fig. 2:**
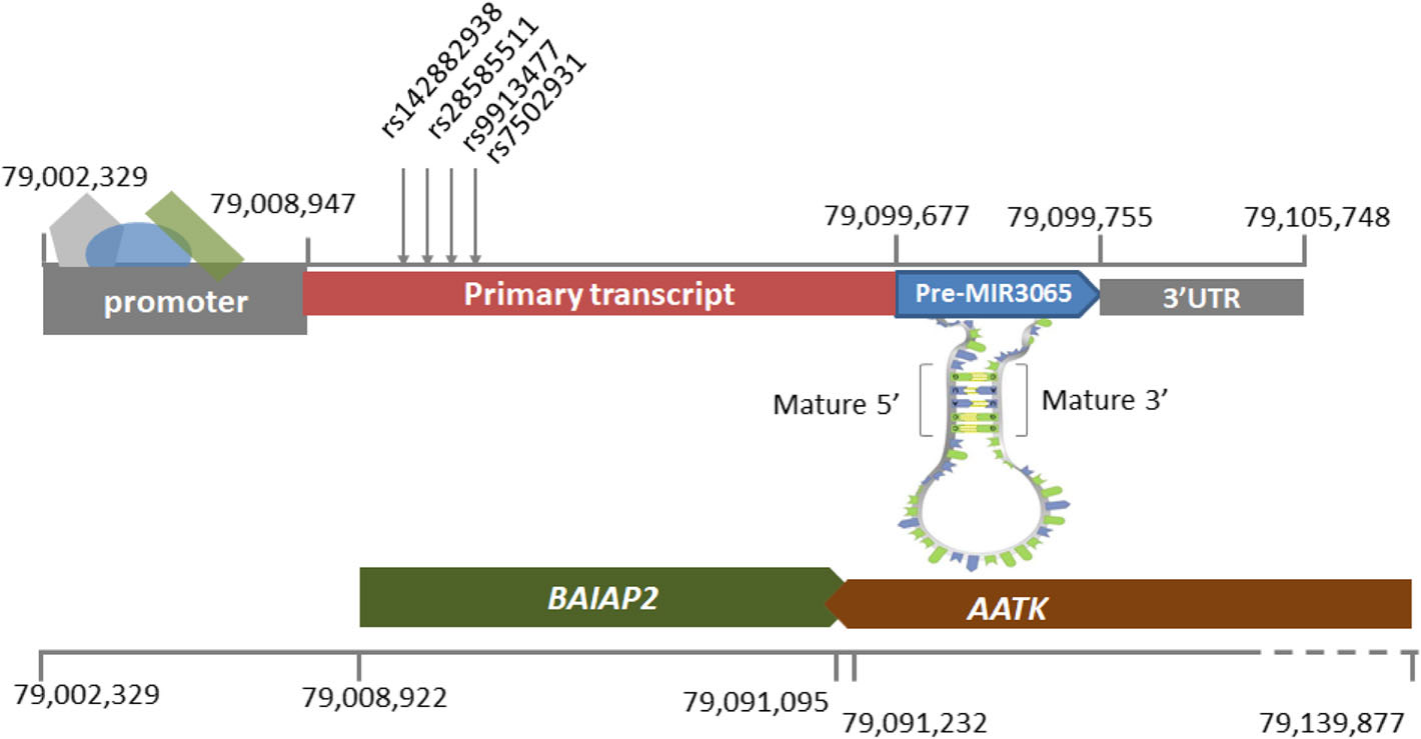
Chromosome 17 position GRCh37/hg19: 79,008,947–79,105,748, encompassing breast cancer-associated SNPs, defined promoter, primary, precursor, mature and 3’-UTR, miR-3065 gene regions and other overlapping coding genes. Note: *BAIAP2* and *AATK* are transcribed in opposite directions. Additionally, *AATK* contains three more miRNAs in close proximity to miR-3065: miR-657, miR-338 and miR-1250

Given their genomic location within the intron of *BAIAP2* and pri-*MIR3065* sequence, these four SNPs have the potential to impact *BAIAP2* expression, *BAIAP2* gene intron 1 binding proteins, and/or *MIR3065* biogenesis. Mature *MIR3065* resides in a gene adjacent to *BAIAP2* known as apoptosis-associated tyrosine kinase (*AATK*) where it is located in the seventh intron and is transcribed in the opposite direction from its host gene. In this gene (*AATK*) and miRNA-rich region, mature *MIR3065* and mature *MIR338* share the same genomic location but are transcribed from opposite DNA strands (Fig. 2) [44]. This critical miRNA sequence region is highly conserved across species [44].

To better understand the implications of inherited susceptibility to BC that may involve *BAIAP2*, we examined expression of this gene in human tissues using data from the Genotype-Tissue Expression (GTEx) project portal version 6 [45]. Human brain-specific angiogenesis inhibitor 1-associated protein 2 (*BAIAP2*) demonstrates a range of expression across various human tissues including brain and breast [45] (Additional file 2: Figure S1). Using data from the Human Protein Atlas project that includes immunochemistry results on 83 different normal cell types from 44 tissue types, we note that moderate *BAIAP2* protein expression is observed in human breast tissue when compared with other normal tissue types [46, 47]. Furthermore, when examining RNA sequencing gene expression of *BAIAP2* in 47 invasive breast carcinoma cell lines from the Cancer Cell Line Encyclopedia [48], we note differential expression with the highest levels (10-fold or more) of *BAIAP2* occurring in four cell lines: EFM-192A, HCC1937, HCC202, and ZR-75-30. Of these four cell lines, two are derived from metastatic sites, with one of these from an African-American woman, the other Caucasian. Of the remaining two cell lines (HCC1937, HCC202), both are from Caucasian women, from primary ductal carcinoma, are ER and PR negative, p53 mutation negative, and positive for EFP2 and CK19 expression; however, they differed in BRCA1 mutation and HER2 status.

The 17q25.3 region containing the top four BC-associated SNPs is extensively marked in the human mammary epithelial cell (HMEC) line by regulatory chromatin states from DNase and histones H3K27ac and H3K4me1, reflecting a number of active promoter and enhancer sequences in the region [44, 49–54]. Furthermore, a number of regulatory sequence motifs (e.g., sequence-specific binding sites for transcription factors) located within intron 1 of *BAIAP2* are altered by these SNPs. Specifically rs9913477 alters regulatory motifs for CDP1 and SOX3 binding while rs7502931 alters a regulatory motif for *ZNF143* [55]. No expression quantitative trait loci (eQTL) were identified in GTEx for any of the four top SNPs [45].

Several epidemiological studies, including both admixture mapping and association analysis of the insulin-related pathway, have examined the 17q region for association with BC in AMBER. A recently published genome-wide case-only admixture scan using 2624 AIMs in the AMBER consortium identified a novel region of excess African ancestry associated with BC risk at 17q25.1 (confirmed in a case-control admixture analysis in the same consortium) [56]. In this admixture scan, AIM rs496948172 provided the largest *Z* score and marked a wide 17q25.1 region of approximately 4.6 Mb where *Z* scores remained above 3.7 indicating excess African ancestry associated with BC. Elevated *Z* scores of 2.4 for this case analysis extend into the 17q25.3 region, where the top hit for the current association analysis (rs9913477) is approximately 1 Mb from the top admixture hit (rs4969481), although LD between these variants is limited (D’ = 0.19, *r*^2^ = 0.0009) based on AFR 1000 Genomes reference panel [57]. Thus, the region of excess African ancestry associated with BC marked by an AIM at 17q25.1 could include the more distal 17q25.3 region as well, providing evidence that variation in this genomic region associated with BC may contribute to disparity in risk. A second AMBER gene-based analysis of 184 genes in the insulin/insulin-like growth factor, leptin, and growth hormone pathways identified *BAIP2* and *CALM2*, and *AIAP2* and *CSNK2A1* as the most significant gene associations (gene-based *p* ≤ 0.01) with both overall and ER positive BC, respectively [58]. Thus, both admixture and insulin pathway-specific association analyses in AMBER provide suggestive evidence of an association with overall and ER-positive BC in the 17q21–25 region. However, due to the relatively less dense SNP set used to evaluate this genomic region in the admixture scan and the less granular nature of the gene-based insulin pathway analysis, neither identified the specific set of four miRNA-associated SNPs localizing a statistically significant association with BC reported here. Additionally, each study strategy for multiple test correction varied in accordance with its statistical methods, with the admixture analysis using a more conservative Bonferroni correction, the gene-based method utilizing a gene-level correction factor, and our current study using a false discovery rate.

A recently published epidemiological study examining miRNA genes and BC among women of African ancestry found fourteen miRNA SNPs associated with overall BC risk at the significance level of 0.05 [29]. Included among these SNPs was rs73410309 within the precursor sequence of *MIR4739* located on chromosome 17q25.3 (OR = 1.1; *p* = 0.039), which is approximately 1.5 Mb from our top hit in *MIR3065* (rs9913477) and not in high LD with this SNP (D’ = 0.007 and *R*^2^ = 0.0) based on AFR 1000 Genomes reference panel [57]. This study was restricted to SNPs within miRNA precursor and mature sequences and thus would not have included the SNP in the primary miRNA sequence identified in our study, but highlights the potential role for miRNA SNPs in BC risk among women of African ancestry in this genomic region.

While no GWAS hits have been reported in the 17q25.3 region, this region has been implicated in several studies of BC tissues where recurrent gain of this genomic region is associated with subtype and recurrence [59, 60]. Gene expression studies of 17q25.3 have identified significant overexpression of 17q25.3 genes in *BRCA1* mutated triple-negative breast cancer (TNBC) as compared with *BRCA1* nonmutated TNBC [59], highlighting the important role that overexpressed sets of genes in this region may play in BC. Given the major role that miRNAs play in global gene regulation it is possible that, even in the absence of a copy number gain, abnormal expression of miRNA genes intended to suppress expression across multiple oncogenes could lead to similar upregulation of sets of genes in this region with similar BC effect. Studies of higher-order chromatin organization have identified regional epigenetic regulation (RER) in breast tumors and BC cell lines that are independent of copy number [61], where 26 regions of coordinate expression were identified between breast tumors and BC cell lines with nine RER showing upregulated gene expression relative to normal breast tissue. Included among these upregulated regions was a 0.9-Mb 17q25.1 region with correlated expression of *KCTD2*, *GGA3*, *MRPS7*, and *GRB2*, and a 0.58-Mb 17q25.3 region (approximately 900 kb from the four associated SNPs reported here) with correlated expression of *STRA13*, *RFNG*, *CSNK1D*, and *SECTM1*.

Perhaps the most compelling support implicating *MIR3065* and BC comes from a recent study by Perrson et al. in which NGS expression analysis in paired normal and breast tumor tissue demonstrates a strikingly disparate expression pattern for *MIR3065* [18]. Among the 361 newly NGS identified miRNA precursors, tumor identity was defined by differences in expression level of a large and common set of miRNAs rather than tissue specific expression [18]. While tissue *MIR3065* expression was highest in breast tumors in a panel of nine human tissues, both lung and placenta demonstrated the next highest expression levels. Similar to previous studies, through BAC array comparative genomic hybridization (CGH), *MIR3065* was also identified as a gene encoded in a region with high-level genomic amplification in luminal B, ERBB2/HER2-positive, ER positive, and ER negative subtypes. Among TargetScan’s (release 6.2, June 2012) *MIR3065* gene top 15 predicted gene targets are the top hit AT-rich interaction domain 4B, *ARID4B* (*alias BRCAA1, breast cancer antigen epitope-1*) and *RAB22A*, a member of the RAS oncogene family of small GTPases involved in signal transduction [62–65]. Immunohistochemically, *ARID4B/BRCAA1* was expressed in 65% of BC specimens but not in noncancerous tissues. and expression was closely associated with ER- and PR-positive status [66]. BC patients also had significantly higher titers of this epitope than healthy donors (*p* < 0.001). Given that two of the top predicted targets are likely oncogenes, it is possible that the role of *MIR3065* is to suppress expression of these oncogenes. It is also possible that the pri-*MIR3065* SNP associated with BC (or a SNP in LD) impairs *MIR3065* processing, leading to lower levels of mature *MIR3065* and reduced inhibition of these oncogenes. Of course, specific gene targets of this new miRNA are not yet fully known and new information may emerge as target prediction algorithms improve and functional data become available. For example, in addition to supporting the potential role of *MIR3065* in BC, Perrson et al. also uncovered a new miRNA in a very well-studied region within the intron of *ERBB2/HER2*, a major predictive marker in BC [18]. This discovery highlights the importance of evaluating genomic regulation beyond the protein coding gene level to examine the major role that noncoding genes, such as miRNAs, may play in cancer development and heterogeneity. These insights will prove invaluable in our understanding of disease development, identifying at-risk populations and providing targets for cancer treatment.

Although this study was limited in scope to miRNAs with SNPs represented or imputed from the Illumina Human Exome BeadChip v1.1 and AMBER custom content (and thus only surveys one-third of all SNPS in the miRBase), as well as by miRNAs with predicted primary sequence from six cell lines, this study is one of the largest evaluations of miRNAs for association with BC. Moreover, it is the largest investigation among African-American women with BC annotated for subtype. Furthermore, predicted boundaries of primary transcripts at both 5′ and 3′ ends were extended from the start of the H3K4me3 peak at the 5′ end (which is often upstream from the actual transcription start site (TSS)) and through the end of the H3K79me2 or H3K36me3 signal (which may or may not be downstream of the transcription termination site). Thus, SNPs defined as retained within the primary transcript may reside just upstream in the miRNA promoter region or may reside just downstream beyond the 3′ end of the primary transcript, thus potentially altering our interpretation of function. Specifically, we predicted that the BC-associated SNPs identified may affect miRNA processing; however, if in fact they reside in the promoter regions, they may influence miRNA expression through other mechanisms. More experimental validation of discrete TSS and end sites is needed for the majority of known miRNAs. Additionally, while this is one of the largest populations of African-Americans with BC examined for miRNA gene association, the subtype analyses remain underpowered for the genetic effect sizes anticipated.

Despite these limitations, this study provides valuable new information about the relationship between numerous miRNA genes and BC in an understudied population, African-American women. It emphasizes the complexity of SNP association analyses and interpretation of function in gene-dense regions, and also the complex interplay of evidence from studies of coding genes, copy number variation, epigenetic regulation, and admixture mapping in an important chromosomal region associated with BC. Functional assessment of the BC-associated SNPs in *BAIAP2* and *MIR3065* are needed to identify the potential molecular mechanism behind their association with BC risk, in particular the risk of ER positive BC, the most common subtype. Larger studies of African-American women are needed to address subtype-specific biology and genetics, including those related to miRNAs.

## Conclusions

This study reports a novel BC signal within an 8.6-kb locus on chromosome 17q25.3, where germline genetic variation is associated with overall and ER positive BC risk among African-American women. This complex and gene-dense region contains *BAIAP2*, a protein-coding gene, and *MIR3065*, an important nonprotein coding regulatory gene, which may play key roles in BC development and heterogeneity among AA women. An understanding of the potentially functional implications of variation in these genes is necessary and may uncover important genetic risk factors and mechanisms for BC in general and, more specifically, for ER positive BC, the most common subtype. Understanding risk factors and mechanisms for BC may lead to improved screening, risk stratification, and novel treatments.

## Additional files

Additional file 1: **Table S1**. Association of the top seven miRNA SNPs from the full analyses with *p* < 5 × 10^−6^ in case versus control and case-only subtype analyses. (XLSX 72 kb)

Additional file 2: **Figure S1**. *BAIAP2* gene expression (from Gene-Tissue Expression project, GTEx) in human tissues [46]. (DOCX 193 kb)

## Abbreviations

AA: African-American
AIM: Ancestry informative marker
AMBER: African American Breast Cancer Epidemiology and Risk
BC: Breast cancer
BWHS: Black Women’s Health Study
CBCS: Carolina Breast Cancer Study
CGH: Comparative genomic hybridization
ChIP-seq: Chromatin immunoprecipitation and massively parallel DNA sequencing
CI: Confidence interval
CIDR: Center for Inherited Disease Research
DCIS: Ductal carcinoma in situ
ENCODE: Encyclopedia of DNA Elements
eQTL: Expression quantitative trait loci
ER: Estrogen receptor
FDR: False discovery rate
GTEx: Genotype-Tissue Expression
GWAS: Genome-wide association study
HER2: Human epidermal growth factor receptor 2
HMEC: Human mammary epithelial cell
kb: Kilobase
LD: Linkage disequilibrium
MAF: minor allele frequencies
Mb: Megabase
MEC: Multiethnic Cohort
MEGA: Multi-Ethnic Genotyping Array
miRNA: MicroRNA
mRNA: Messenger RNA
NGS: Next-generation sequencing
nt: Nucleotide
oncomiR: Oncogenic miRNA
OR: Odds ratio
PR: Progesterone receptor
pre-miRNA: Precursor microRNA
pri-miRNA: Primary microRNA transcript
QC: Quality control
RER: Regional epigenetic regulation
RISC: RNA-induced silencing complex
SNPs: Single nucleotide polymorphisms
TNBC: Triple-negative breast cancer
TSS: Transcription start site
UTR: untranslated region
WCHS: Women’s Circle of Health Study
