# Supplementary figures and images for: A survey of microRNA single nucleotide polymorphisms identifies novel breast cancer susceptibility loci in a case-control, population-based study of African-American women
Source: Breast Cancer Res. 2018 Jun 5;20:45. doi: 10.1186/s13058-018-0964-4 (PMC5989404; doi:10.1186/s13058-018-0964-4)

**
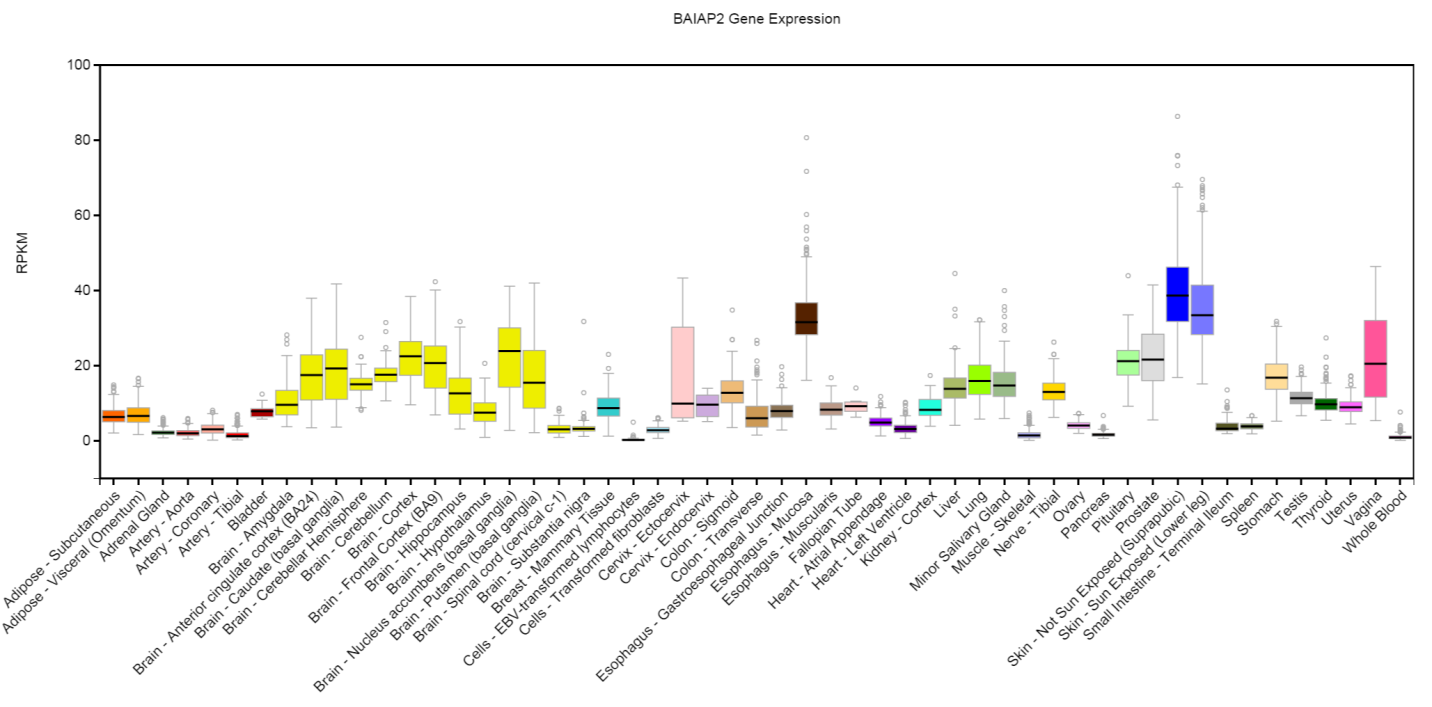
**

Figure S1.*BAIAP2* gene expression (from Gene-Tissue Expression project, GTEx) in human tissues (46).

Supplement: Supplementary file 2 — Figure S1. BAIAP2 gene expression (from Gene-Tissue Expression project, GTEx) in human tissues [46]. (DOCX 193 kb) [file 13058_2018_964_MOESM2_ESM.docx]
